# BEFRIENding for Depression, Anxiety and Social support in older adults living in Australian residential aged care facilities (BEFRIENDAS): randomised controlled trial protocol

**DOI:** 10.1186/s12877-021-02233-7

**Published:** 2021-05-12

**Authors:** Colleen Doyle, Sunil Bhar, Christina Bryant, Briony Dow, David Dunt, George Mnatzaganian, Daniel O’Connor, Julie Ratcliffe, Emily You, Anne-Marie Bagnall, Georgia Major, Robin Harper, Marcia Fearn

**Affiliations:** 1 Aged Care Division, National Ageing Research Institute, Poplar Road, Parkville, 3052 Australia; 2 Department of Psychological Sciences, Swinburne University, John Street, Hawthorn, 3122 Australia; 3 School of Nursing and Midwifery, Deakin University, Burwood Highway, Burwood, 3125 Australia; 4 Melbourne School of Psychological Sciences, Faculty of Medicine, Dentistry and Health Sciences, The University of Melbourne, Grattan Street, Parkville, 3010 Australia; 5 School of Population and Global Health, The University of Melbourne, Grattan Street, Parkville, 3010 Australia; 6 La Trobe Rural Health School, La Trobe University, Bendigo, 3552 Australia; 7 Faculty of Medicine, Nursing and Health Sciences, Monash University, Clayton, 3800 Australia; 8 Caring Futures Institute, Flinders University, Sturt Road, Bedford Park, 5042 Australia; 9 Academic Unit for Psychiatry of Old Age, The University of Melbourne, Poplar Road, Parkville, 3052 Australia; 10 Leeds-Beckett University, Leeds, LS1 3HE United Kingdom

**Keywords:** Depression, Anxiety, Loneliness, Befriending, Volunteers

## Abstract

**Background:**

This protocol describes an ongoing study of the impact of befriending on depression, anxiety and loneliness in older people living in residential aged care facilities in Australia. While systematic reviews of befriending have indicated positive benefits of befriending for people in a range of ages and settings, there have been no randomised controlled trials (RCTs) of befriending for older people living in residential aged care with depression and no studies of the cost effectiveness of befriending in residential aged care facilities (RACFs) in Australia.

**Methods and analysis:**

We are conducting a single blind pragmatic RCT comparing two groups of older people living in RACFs, one receiving an intervention consisting of weekly befriending for 4 months from a trained volunteer and the other receiving treatment as usual. Participants undergo eligibility screening for depression (GDS-15 ≥ 4) and cognitive impairment (GPCog ≥ 4) and assessments at three measurement time points: baseline prior to randomisation, 2 months post-baseline and 4 months post-baseline. The primary outcome measure is depression, and secondary outcome measures are anxiety, loneliness, social isolation and quality of life. The economic evaluation will take the form of a cost-utility analysis based on the outcome of quality of life. The primary and secondary outcomes will be analysed using negative binomial and logistic regressions utilizing the Generalised Estimating Equations approach.

**Discussion:**

To our knowledge, this is the first RCT evaluating the effectiveness of befriending on older people with depression living in residential aged care. It is expected that the befriending intervention will reduce the severity of depression symptoms experienced by older people living in residential aged care. If the intervention proves effective it may be incorporated into volunteer training programs and adopted as a way of supporting older people’s mental health.

**Trial registration:**

Trial registered with the Australian and New Zealand Clinical Trial Registry (ANZCTR) Number: ACTRN12619000676112, registered 06/05/2019 – retrospectively registered.

**Supplementary Information:**

The online version contains supplementary material available at 10.1186/s12877-021-02233-7.

## Background

Depression symptoms are experienced by many people living in residential aged care facilities (RACFs, equivalent to UK care homes and US nursing homes or long term care homes). Up to 53% of people living in Australian RACFs have been shown to have significant symptoms of depression [1, 2]. Internationally, the prevalence of depressive symptoms in people living in RACFs has been estimated to range between 11 and 78% [3]. Major depressive disorder has been estimated at 10–14.4%, which is higher than that reported within community dwelling older adults [3–6]. Despite living in a communal environment, many people living in RACFs are socially isolated [7]. Social isolation and loneliness are closely associated with symptoms of depression [8] and a recent qualitative study found that residents in RACFs attributed their sadness to loss of health and functional ability, and feeling alone in a crowd [9].

One reason for the higher prevalence of depression in RACFs may be that staff are not trained to manage depression, but studies of training staff in the detection and management of depression have met with short-term success and little sustainable change [10]. Evidence-based recommendations for the management of depression include anti-depressants or other medical treatments, psycho-education, social support, and psychological interventions [11]. In general the prescription of psychotropic medication is high among RACF residents [12, 13], but older adults have expressed concerns about using anti-depressants due to side-effects, stigma, fear of addiction and prevention of natural sadness [14]. On the other hand, psychological services can be difficult for RACF residents to access in Australia [15], as psychologists are not typically employed in Australia in such settings [16]. Furthermore, residents who are depressed or anxious are less likely to want to join in lifestyle activities by the very nature of their condition [11].

Befriending has been recommended in the National Institute for Health and Care Excellence (NICE) guidelines to assist people with moderate to severe depression who would benefit from additional social support and for those with subclinical persistent depression although there have been no studies of befriending for RACF residents [11]. Befriending is generally defined as non-directive emotional and social support which is typically administered by non-health professionals. Befriending conversations avoid health-related matters and focus on enjoyable topics of interest to both parties. The NICE guidelines recommend at least weekly befriending sessions for 2 to 6 months to gain benefit. Telephone befriending has been shown to help recipients gain confidence, re-engage with the community and become socially active again [17].

A review by Siette et al. [18] included 14 papers that focused on befriending, however none evaluated befriending in RACFs. According to that review, the training, intensity and the frequency of befriending conversations all varied in the studies reviewed [18]. In previous befriending studies, conversations were delivered one-on-one, face-to-face or by phone but could also be delivered in group support settings [19]. To date there is little evidence for the effectiveness of befriending via video calls [20]. Therefore Siette and colleagues found mixed results and recommended more specific eligibility criteria and better definitions for future studies. Despite loose definitions, befriending has been recommended as a useful complement to clinical treatments because it has potential to improve mental health outcomes. It also may be a cost effective complement to clinical treatment when administered by volunteers, although formal cost-effectiveness studies have not been published to date.

Befriending conversations can be delivered by volunteers. Research on the role of volunteers in health services has consistently shown positive effects for volunteers and older recipients of the service [21] including through: assisting with activities for socially isolated residents in aged care [22, 23]; engaging in social visits with residents in aged care [24]; and providing social support over the phone for people in the community with depression, anxiety and comorbid physical health problems [25]. When provided by volunteers, befriending has been found to significantly reduce depression symptoms in carers, those with a chronic illness, and the socially isolated, but again studies in RACF settings have not been published to date [26].

**Fig. 1:**
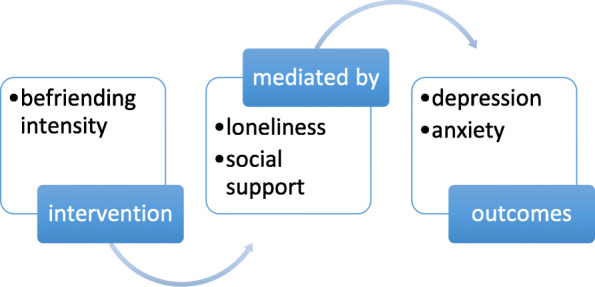
Hypothesized mediating variables between befriending and depression

The primary aim of the BEFRIENDAS study is to test the effectiveness of befriending in relieving depression symptoms experienced by older adults living in RACFs compared with a control group receiving usual care. Secondary aims are to test its effectiveness in reducing symptoms of loneliness, anxiety, social isolation and improving quality of life. A conceptual framework or explanation for the mechanism underlying befriending is lacking in the literature, but we hypothesise that the impact of befriending on depression and anxiety is mediated by social support and reductions in loneliness (see Fig. 1).

## Methods and analysis

This protocol adheres to the SPIRIT 2013 checklist for recommended items to address in a clinical trial protocol. An additional file shows this in more detail (see Additional file 1).

### Study design

**Fig. 2:**
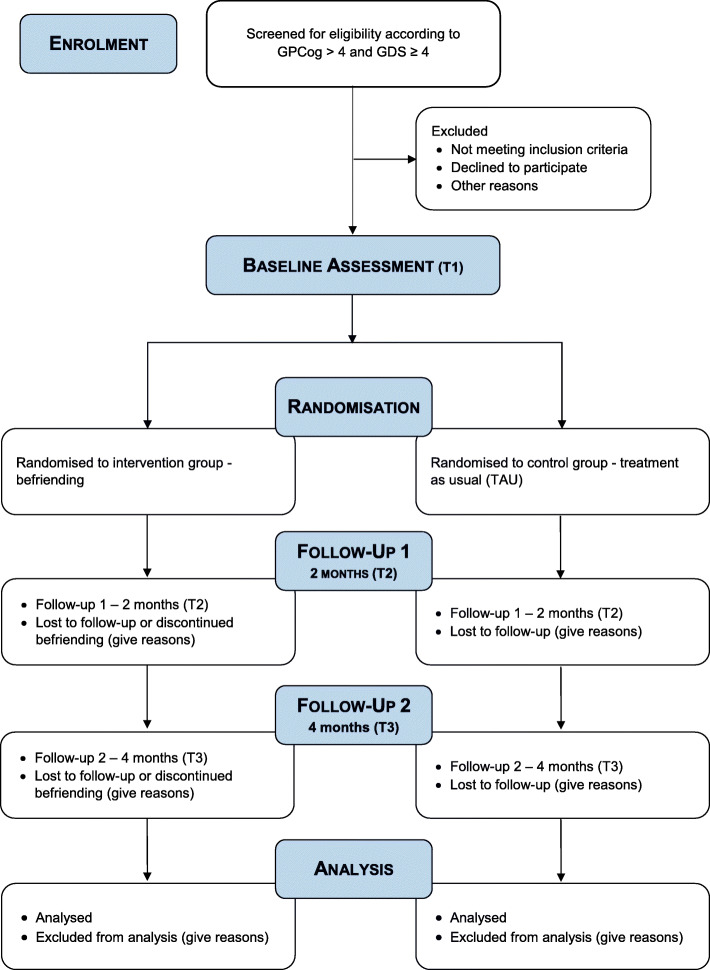
Flow chart of participant flow

The BEFRIENDAS study is an ongoing single blind pragmatic RCT comparing two groups of older people living in RACFs, one receiving weekly befriending for 4 months from a trained volunteer and the other receiving treatment as usual (TAU). As few previous RCTs of befriending have been conducted, TAU is an appropriate comparator. A clustered design is not necessary because the intervention is to be delivered at the individual level and not the RACF level. Furthermore, there is no evidence that RACFs have different policies on treatment of depression. Group allocation is blinded to the assessors but necessarily un-blinded to the participant. Participants undergo eligibility screening and assessments at three measurement time points: baseline prior to randomisation (T1), 2 months after the intervention starts (T2) and at the conclusion of the intervention 4 months post-baseline (T3). Figure 2 presents the flow of the participants through the study.

### Participants

Participants are people over the age of 65 years living in RACFs in Australia. RACFs are a convenience sample recruited through advertisement in industry publications, word of mouth and social media. Participants in individual RACFs are identified and recruited with the assistance of a clinical care manager, lifestyle coordinator or other staff member within the RACF. The staff identify all those eligible to participate in the study using initial eligibility criteria provided below and then approach residents to gauge their interest in participating. Following instructions provided by the research team, including allowing residents to view an ‘introduction to the project’ video, the staff member explains the study to potential participants in a non-coercive way. If the resident assents, the research assistant meets with them either face-to-face or remotely via telephone or video call to conduct further eligibility screening, confirms eligibility, provides additional information about the study and gains written informed consent to proceed. All consent is provided by participants not by proxies.

#### Initial eligibility criteria used by staff to identify eligible residents

1. Cornell Scale for Depression in Dementia (CSDD) [27] score of 8 or greater as assessed by RACF staff (no longer than 6 months prior) or
2. Psychogeriatric Assessment Scale – Cognitive Impairment Scale (PAS-CIS) [28] score equal to or less than 10, as assessed by RACF staff (no longer than 6 months prior) or
3. Clinical judgement of clinical care manager indicating significant depression or anxiety symptoms.

#### Further eligibility screen conducted by research assistants to confirm eligibility

1. Geriatric Depression Scale 15-item (GDS 15) [29] score of 4 or greater as assessed by the research assistant.
2. General Practitioner Assessment of Cognition (GPCOG) [30] score of 4 or greater as assessed by the research assistant.

Residents who are unable to give informed consent, have progressive, unstable medical or neurological illness, a diagnosis of dementia or who are under the age of 65 years are excluded from the project.

Volunteers administer the befriending intervention and participate in qualitative evaluation of the experience. Volunteers are a convenience sample recruited via volunteer recruitment websites, social media advertisement, word of mouth and through advertisements in industry newsletters. Volunteers are eligible to participate if:

- over 18 years and have a valid police check
- have an up to date influenza immunisation
- assessed as suitable (according to availability, conversation skills and attitudes) in an interview with the volunteer coordinator
- available for at least 6 months of volunteering
- able to travel to the RACF where appropriate
- willing to participate in training and ongoing supervision.

#### Patient and public involvement

The befriending intervention is a manualised and structured intervention (manuals available from corresponding author). The topics of conversation are guided by the resident participants and volunteers. A project advisory group will be established to guide the dissemination of the findings and to advise on how the befriending model can be translated into practice in RACFs. This advisory group will consist of staff representatives from RACFs, residents from RACFs and family members of residents from RACFs.

### Sample size

We are aiming for a clinically significant change of greater than two points in the GDS. Based on the average effect size of 0.27 for short term befriending outcome studies of mostly older adults with depression in primary care settings, it is estimated that with data collection spread over a three-year period, and allowing for a higher than usual dropout rate of 20% given low life expectancy among RACF residents, recruiting 172 participants per study arm (total of 344 participants) would achieve 80% power, with a statistical significance of 0.05. It is anticipated that each volunteer recruited may only befriend one participant, so a total of over 500 volunteers will be recruited throughout the study. Recruitment strategies to achieve the sample include wide ongoing advertisement and presentations in industry meetings and publications, monitoring quotas and widening locations where needed.

### Randomisation

Following the baseline assessment, participants are randomly allocated to the befriending intervention group or the TAU control group. Relevant concomitant care and interventions such as psychological interventions or anti-depressants are noted and considered treatment as usual. Randomisation is managed by a trial statistician who is not directly involved in the study. A computer-generated random sequence available to the trial statistician, but no other research staff, is used to produce random allocation using a randomised permuted block design with block size of two in order to maintain approximately balanced randomisation throughout the trial. Randomisation is provided to an un-blinded researcher via email to verify that the correct allocation has taken place. The un-blinded researcher notifies the participants about their allocation and arranges for the intervention to begin. Research assistants conducting the assessments are blinded to group allocation.

### Intervention

The intervention consists of weekly befriending sessions from a trained volunteer for a four-month period, and is provided in addition to usual care. Befriending is defined as “acting or becoming a friend to (someone), especially when they are in need of help or support” [31]. Befriending conversations consist of neutral topics of interest to both parties, including discussions around hobbies, family, neutral news topics, and other topics to be negotiated between the two parties and tailored to individual interests. Volunteers undergo standardised training on how to conduct the intervention. A manual is provided for volunteers to refer to throughout their befriending experience (available on request). The training is delivered either face-to-face in small interactive groups or via online modules and video conference. Topics covered in the training session include: what to expect when visiting a RACF; characteristics of residents in RACFs; how to conduct befriending conversations; topics to avoid; what to do if a resident becomes upset; how to manage indications of elder abuse; key symptoms of depression and anxiety; how to communicate with someone with mild cognitive impairment; how much personal information to disclose; and how to say goodbye at the end of the intervention. The training includes reflective questions on the content and allows for individual variation in experience of RACF settings. Responses to the training are evaluated using pre and post questionnaires and ongoing refresher tests. Volunteers participate in focus groups to discuss their response to their befriending experiences.

At each RACF, befriending sessions are held face-to-face in a mutually agreed private setting or remotely via phone or video call depending on local biosecurity guidelines during the COVID-19 pandemic. Sessions may switch from one format to another depending on local circumstances. There is no required minimum length of time for each befriending session, however it is anticipated that each session will last for approximately 30–60 min and not more than 2 hours. The control group receives usual care during the assessment period, and participants are offered a volunteer befriender at the completion of their four-month assessment period, but the impact of these sessions will not be evaluated as part of the study.

To maintain fidelity of the intervention, volunteers undergo refresher quizzes and have weekly mentoring and supervision with MF, GMajor or CD. These may be individual or group based and conducted via email, phone or video conference. Volunteers audio record their befriending conversations to allow random monitoring of conversation recordings for quality and integrity analyses. A random 5% sample of recordings is listened to bi-monthly by two researchers to judge that the topics discussed are not health-related or therapeutic. Volunteers make notes of all befriending sessions in a standardised template, recording information such as the length of the session, as well as information about topics that were discussed and any issues that arose. Conversation notes on the topics of conversation are transcribed and analysed thematically to check that the intervention is being adhered to. Other study measures of the fidelity and acceptability of the intervention include the percent of pairings achieving full befriending (ie at least 12 sessions), reasons for failure where relevant, resident ratings of satisfaction with intervention and resident perceptions of the befriending intervention.

If volunteers need to leave the study before the end of their befriending intervention they will be replaced with other trained volunteers for the residents to complete their intervention period.

### Outcome measurements

#### Primary outcome measure

The primary outcome in the study is depression, as measured by the GDS 15 [29], a reliable and valid screening tool for depression in older people. It comprises “Yes/No” questions about how the participant has felt over the past week. Of the 15 items, 10 indicate the presence of depression when answered positively and five indicate depression when answered negatively. Higher absolute total scores indicate increased depressive symptoms are present.

#### Secondary outcome measures

Secondary outcomes are anxiety, loneliness, social isolation and quality of life. These outcome measures will capture negative as well as positive effects. Anxiety is measured by the Geriatric Anxiety Inventory (GAI 20-item) [32], consisting of 20 “Agree/Disagree” items designed to assess typical common anxiety symptoms in older adults. Loneliness is measured by the UCLA Loneliness Scale [33], a 20-item scale designed to measure a participant’s subjective feelings of loneliness as well as feelings of social isolation. Participants rate each item as either Always (“I always feel this way”), Sometimes (“I sometimes feel this way”), Rarely (“I rarely feel this way”) or Never (“I never feel this way”). Social isolation is measured by The Lubben Social Network Scale-6 [34]. This scale is designed to gauge social isolation in older adults by measuring the number and frequency of social contacts with friends and family members. Health related quality of life is measured by the EQ-5D-5L [35] which comprises five dimensions: mobility, self-care, usual activities, pain/discomfort and anxiety/depression. Each dimension has five levels: no problems, slight problems, moderate problems, severe problems and extreme problems. The patient’s self-rated health is recorded on a vertical visual scale, where the endpoints are labelled ‘The best health you can imagine’ and ‘The worst health you can imagine’.

#### Descriptive measures

Demographic information about the participants is collected, including gender, date of birth, county of birth, date of admission to the residence, medical conditions and comorbidities as measured by the Charlson Comorbidity Index [36], activities of daily living capabilities as measured by the Aged Care Funding Instrument (ACFI) Activities of Daily Living domain which consists of questions about nutrition, mobility, personal hygiene, toileting and continence [37], marital/relationship status, highest education level and ethnic and gender diversity details.

#### Economic evaluation

An economic evaluation will be conducted to assess the cost effectiveness of the befriending intervention. The economic evaluation will take the form of a cost-utility analysis based on the outcome of quality of life as measured by the EQ-5D-5L. Health service utilisation data on the frequency and duration of access to primary care, emergency department visits, overnight hospital stays, psychiatrists, psychologists and other mental health practitioners are obtained from RACF records. Medications are obtained from the RACF records.

#### Qualitative measures

Qualitative methods are being used to increase our depth of understanding about befriending. Participants in the intervention arm are invited to participate in a short semi-structured interview at the end of the intervention. Participants are asked questions about their initial reactions to befriending, change in perception over time, benefits and disadvantages of befriending, matching of personalities, logistical issues such as frequency and intensity of visits and the format of the befriending. All volunteers are invited to participate in focus groups or individual interviews to gauge their perception of the befriending experience.

### Data collection and management

Data collection is performed by blinded research assistants trained to administer the outcome measures in a structured interview, with the exception of some data available from the resident’s RACF records, which is provided by the RACF staff. Inter-rater reliability between research assistants is tested regularly for the GDS 15, GAI 20-item and UCLA Loneliness Scale using the Kappa statistic to monitor consistency. Discrepancy in assessments (kappa less than 0.4) is discussed and consistent probing statements are used for each outcome measure to maximise consistency across time and between research assistants.

The qualitative interviews are digitally recorded and transcribed verbatim by a professional transcription service. A data monitoring committee is not needed because the trial is not testing a drug or device and any safety concerns associated with the trial have been reviewed by the ethics committee. Participants are informed that they can withdraw from the study at any time and any incomplete data is retained by the project team.

#### Assessment timepoints

Table 1 shows the timepoints when each outcome measure is conducted: at baseline assessment (T1), 2 months post commencement of intervention (T2) and follow-up assessment 4 months post commencement (T3).

**Table 1:** Measures and assessment timepoints

Measure/Timepoint	Screen	T1 Baseline	T2 2 months post baseline	T3 4 months post baseline
GDS 15	✓		✓	✓
CSDD (from ACFI)^a^	✓			
PAS-CIS (from ACFI)^a^	✓			
GPCOG	✓			
Demographic data		✓		
GAI 20 item		✓	✓	✓
EQ-5D-5L		✓	✓	✓
UCLA Loneliness Scale		✓	✓	✓
Lubben Social Network Scale-6		✓		✓
Health service usage		✓		✓
Charlson comorbidity index		✓		✓
Medications		✓		✓
Satisfaction with befriending (qualitative data)				✓
Perceptions of befriending (qualitative data)				✓

^a^Provided to research team after resident provides consent to be involved in the study

#### Data storage

All hard copy data are stored in a coded (re-identifiable) form in a locked filing cabinet at the National Ageing Research Institute. All electronic data are coded (re-identifiable) and stored on a password-protected server at the National Ageing Research Institute. Trial data are only accessible by the research team, and the National Ageing Research Institute Research Governance Officer if necessary.

### Data analysis

No interim analyses to inform stopping guidelines will be required. The balanced characteristics between the intervention and control arms will be assessed by comparison of all baseline characteristics between the two study groups using χ^2^ test for dichotomous variables and either ANOVA or Kruskal-Wallis test for continuous data. Comparison of descriptive data will be made between participants who completed the study and those who dropped out to assess differential attrition. The primary and secondary outcomes (change over time in depression, anxiety, loneliness and social isolation) will be analysed using negative binomial and logistic regressions utilizing the Generalised Estimating Equations (GEE) approach, a marginal model in which the effect of covariates on the outcome is averaged over individuals at each point in time and is compared over time [38]. An exchangeable working matrix will be used to account for correlation and dependence between repeated measurements on the same individual over time while adjusting for study covariates.

Data analysis will investigate possible mediating variables explaining any relationship found between befriending and depression symptoms. Possible intervening variables of interest will include baseline depression/anxiety (higher is worse); baseline cognition (higher is better); and intensity of befriending (more befriending sessions are better). For the economic analysis, utility based outcomes, generated from application of the EQ-5D-5L, will be used to calculate the cost per quality-adjusted life-year (QALY) gained. Costs will include staff time spent preparing and producing training materials in addition to staff time spent administering/managing the befriending intervention. Unit costs for the mental health service utilization data will be derived from published fee schedules. Incremental cost effectiveness ratios and their associated confidence intervals will be estimated and cost effectiveness acceptability curves for varying threshold values of cost effectiveness will also be presented. An assessment of the sensitivity of the results obtained to variation in measured resource use, effectiveness and/or unit costs will be undertaken using appropriate one-way and multi-way sensitivity analysis.

A qualitative, phenomenologically based research design will be used to explore the experience of befriending from the perspective of the participants [39] and so to determine the acceptability and feasibility of the intervention. Colaizzi’s phenomenological data analysis steps will be used to describe the phenomena experienced by the participants and by the volunteers [40, 41].

## Discussion

This study protocol presents the design of an ongoing RCT that aims to test the effectiveness of befriending by a volunteer on depression, anxiety and loneliness symptoms in older people living in residential aged care. To our knowledge, this is the first RCT evaluating the effectiveness of befriending on older people with depression living in residential aged care.

The introduction of the CSDD into the ACFI was a change in policy recognising the significance of psychological symptoms among RACF residents in Australia, but few other systematic efforts have addressed the issue. Guidelines around the management of depression, such as the NICE guidelines for the management of depression [11] are well established but not necessarily well implemented in RACFs where there is limited access to psychological services.

The befriending intervention, provided by trained volunteers over a four-month period, may reduce severity of depression experienced by older people living in RACFs, as well as the number of anxiety and loneliness symptoms. The results of this study will provide useful information about the effectiveness of a volunteer befriending program with older people living in RACFs, such as the necessary number of sessions required to have an impact on the resident, the length that the session needs to be and qualitative information on the acceptability of the delivery format, that is face-to-face or remotely via the telephone or video call.

If befriending, as conducted in this trial, proves effective, it is anticipated that befriending can be incorporated into health promotion programs such as the volunteer education program titled “Volunteers enriching older people’s wellbeing” offered by Beyond Blue, a health promotion organisation in Australia dedicated to improving mental health. It is also anticipated that this model may be adopted as a cost effective means of supporting residents with psychological symptoms living in RACFs. It is anticipated that it may be a complement rather than a substitute for other mental health support services. In light of the COVID-19 pandemic and the impact of lockdowns on the mental health of older people living in RACFs, the results of this study will be especially important to inform future models of care of the mental health of frail older people living in residential care [42, 43].

### Dissemination

There will be no limitations to the dissemination of the results. It is anticipated that the results of this research project will be published and/or presented in a variety of forums, including peer reviewed journal publications, conference presentations, aged care industry communications and media releases. In any publication and/or presentation, information will be provided in such a way that participants cannot be identified.

## Supplementary Information

**Additional file 1.** SPIRIT Checklist – checklist of recommended items to address in a clinical trial protocol

## Data Availability

Resultant data sets may be made available upon reasonable request of the corresponding author once the study is concluded.

## Abbreviations

ACFI: Aged Care Funding Instrument
CSDD: Cornell Scale for Depression in Dementia
GAI: Geriatric Anxiety Inventory
GDS: Geriatric Depression Scale
GPCOG: General Practitioner Assessment of Cognition
NICE: National Institute for Health and Care Excellence
PAS-CIS: Psychogeriatric Assessment Scale – Cognitive Impairment Scale
QALY: Quality adjusted life year
RACF: Residential aged care facility
RCT: Randomised controlled trial
TAU: Treatment as usual
