# Correction: BEFRIENding for depression, anxiety and social support in older adults living in Australian residential aged care facilities (BEFRIENDAS): randomised controlled trial protocol

**DOI:** 10.1186/s12877-025-06181-4

**Published:** 2025-07-15

**Authors:** Colleen Doyle, Sunil Bhar, Christina Bryant, Briony Dow, David Dunt, George Mnatzaganian, Daniel O’Connor, Julie Ratcliffe, Emily You, Anne-Marie Bagnall, Georgia Major, Robin Harper, Marcia Fearn

**Affiliations:** 1 Aged Care Division, National Ageing Research Institute, Poplar Road, Parkville, 3052 Australia; 2 Department of Psychological Sciences, Swinburne University, John Street, Hawthorn, 3122 Australia; 3 School of Nursing and Midwifery, Deakin University, Burwood Highway, Burwood, 3125 Australia; 4 Melbourne School of Psychological Sciences, Faculty of Medicine, Dentistry and Health Sciences, The University of Melbourne, Grattan Street, Parkville, 3010 Australia; 5 School of Population and Global Health, The University of Melbourne, Grattan Street, Parkville, 3010 Australia; 6 La Trobe Rural Health School, La Trobe University, Bendigo, 3552 Australia; 7 Faculty of Medicine, Nursing and Health Sciences, Monash University, Clayton, 3800 Australia; 8 Caring Futures Institute, Flinders University, Sturt Road, Bedford Park, 5042 Australia; 9 Academic Unit for Psychiatry of Old Age, The University of Melbourne, Poplar Road, Parkville, 3052 Australia; 10 Leeds-Beckett University, Leeds, LS1 3HE UK

**Correction: BMC Geriatr 21**,** 305 (2021)**

10.1186/s12877-021-02233-7

Following publication of the original article [1], the authors reported incorrect data under the ‘Sample size’ section. The incorrect data ‘**272 participants per study arm (total of 544 participants)**’ should be corrected to ‘**172 participants per study arm (total of 344 participants)**’.

The original article [1] has been corrected.
